# Consequences of Beauty: Effects of Rater Sex and Sexual Orientation on the Visual Exploration and Evaluation of Attractiveness in Real World Scenes

**DOI:** 10.3389/fnhum.2016.00122

**Published:** 2016-03-21

**Authors:** Aleksandra Mitrovic, Pablo P. L. Tinio, Helmut Leder

**Affiliations:** ^1^Department of Basic Psychological Research and Research Methods, Faculty of Psychology, University of ViennaVienna, Austria; ^2^Department of Educational Foundations, Montclair State University, MontclairNJ, USA

**Keywords:** eye movements, facial attractiveness, sexual orientation, mate choice, aesthetics

## Abstract

One of the key behavioral effects of attractiveness is increased visual attention to attractive people. This effect is often explained in terms of evolutionary adaptations, such as attractiveness being an indicator of good health. Other factors could influence this effect. In the present study, we explored the modulating role of sexual orientation on the effects of attractiveness on exploratory visual behavior. Heterosexual and homosexual men and women viewed natural-looking scenes that depicted either two women or two men who varied systematically in levels of attractiveness (based on a pre-study). Participants’ eye movements and attractiveness ratings toward the faces of the depicted people were recorded. The results showed that although attractiveness had the largest influence on participants’ behaviors, participants’ sexual orientations strongly modulated the effects. With the exception of homosexual women, all participant groups looked longer and more often at attractive faces that corresponded with their sexual orientations. Interestingly, heterosexual and homosexual men and homosexual women looked longer and more often at the less attractive face of their non-preferred sex than the less attractive face of their preferred sex, evidence that less attractive faces of the preferred sex might have an aversive character. These findings provide evidence for the important role that sexual orientation plays in guiding visual exploratory behavior and evaluations of the attractiveness of others.

## Introduction

In our daily interactions, we frequently find some individuals more attractive than others. These evaluations of attractiveness are driven by our esthetic sense, which, according to Darwin (1871), evolved to facilitate good mating decisions by drawing us to individuals who are, for example, genetically healthy (see also Dion et al., 1972; Thornhill and Gangestad, 1993, 1996; Thornhill and Grammer, 1999; Senior, 2003; Dissanayake, 2007).

More generally, attractiveness also plays a crucial role in our interactions with people. Attractive faces draw more attention and seem to demand longer looks. Attractive faces bind attention. Evidence for this was provided by Shimojo et al. (2003) who asked participants to look at pairs of isolated faces. Participants then selected which of each pair of faces was more attractive. Participants looked longer at the more attractive faces and this effect became even stronger as the time to select (recorded via key press) the more attractive face approached. The authors interpreted this as a “gaze cascade effect,” with liking increasing because of the longer looks.

Leder et al. (2010) found similar effects of longer and more frequent looks to attractive faces, when faces were embedded in images of real-world scenes. They measured eye movements of participants who freely viewed (no task or decision required) faces that were embedded in images of real world scenes. The scenes consisted of urban street scenes with each scene depicting two same-sex people who each represented one of two levels of attractiveness (attractive and less attractive, according to pre-studies)^1^. Results showed that attractive faces received longer and more frequent looks. These results were corroborated with participants’ subjective ratings: not only were attractive faces rated as more attractive than less attractive faces, but female faces were rated as more attractive than male faces. Furthermore, differences between attractive and less attractive faces were larger for female than male faces, and women provided higher ratings for attractive faces than did men. Interestingly, Leder et al. (2010) also found that women looked longest at attractive female faces.

The above results (e.g., Leder et al., 2010) raise an intriguing question regarding the roles of perceiver’s sex and sexual orientation on visual exploratory behavior. Regarding the former, studies have shown that the perceiver’s sex is indeed important. For instance, attractiveness is not equally important for men and women, and men and women also distribute their attention differently. Alexander and Charles (2009) and Nummenmaa et al. (2012) showed that heterosexual men looked longer at female than male faces; in contrast, heterosexual women distributed their attention more evenly between the sexes. Lykins et al. (2008) as well as Israel and Strassberg (2009) also found that heterosexual men and women looked longer at people of the other sex as compared to people of the same sex. Some authors have additionally reported evidence that attractiveness is generally more important for heterosexual than for homosexual individuals, and that it is more important for heterosexual men and homosexual men than for heterosexual women, and least important for homosexual women (Bailey et al., 1994; Russock, 2011; Ha et al., 2012). All of these studies suggest that the factors sex of the perceiver and sex of the perceived face should both be taken into account in studies of facial attractiveness. These two factors, along with sexual orientation, are central topics in the present study.

Regarding sexual orientation, there are differences between heterosexual and homosexual individuals in much the same way as there are differences between men and women (e.g., that attractiveness is more important for men than for women). Such differences have been found at the neurological level (Swaab and Hofman, 1990; LeVay, 1991; Savic and Lindström, 2008). For example, Kranz and Ishai (2006) and Ishai (2007) reported evidence that certain brain responses associated with reward are determined to a great extent by perceivers’ sexual orientation. They showed pictures of male and female faces to heterosexual and homosexual men and women who then rated the attractiveness of the faces. During the rating task, heterosexual women and homosexual men showed similar brain activity in the thalamus and medial orbitofrontal cortex (measured with functional magnetic resonance imaging) when seeing faces of men; and heterosexual men and homosexual women when seeing faces of women. The orbitofrontal cortex receives input from the thalamus, and the medial orbitofrontal cortex is involved with processing of reward. Other evidence also suggests that faces matching perceivers’ sexual orientation are more important and attract more attention than sexually non-preferred faces. Lippa (2012) found that heterosexual and homosexual men and women rated pictures of the preferred sex as more attractive and looked at them longer. Men did not rate pictures of the non-preferred sex as more attractive as attractiveness increased; women, however, rated both sexes more attractive with increasing attractiveness. Moreover, for homosexual men, durations of looks did not increase with increasing attractiveness of either their preferred or non-preferred sex. Through an eye tracking experiment, Fromberger et al. (2011) also examined the effects of sexual orientation. In their study, heterosexual men looked at various pictures showing men (considered to be non-preferred in a sexual regard because of their sex), boys and girls (considered to be non-preferred in a sexual regard because of their age), and women (considered to be sexually preferred in terms of sex and mating). Results showed that participants looked first, and for longer durations, at the sexually preferred faces. These results further highlight the importance of taking sexual orientation into account when conducting research on the perception of others.

In order to disentangle the various factors involved in facial attractiveness, and its behavioral consequences, we conducted a study that examined how an individual’s sex and sexual orientation influence visual exploratory behavior toward, and evaluations of, faces. A noteworthy difference between heterosexual and homosexual individuals is that for the former group, other sex people represent possibilities for both romantic partnership and reproduction; for the latter group, romantic partnership takes precedence. However, being oriented toward one’s own sex of course does not preclude the desire to have children.

As indicated in the above literature review, looking behavior seems to be a suitable measure of sexual interest or attraction. We therefore used eye tracking in the present study and analyzed looks toward each of two faces that varied in levels of attractiveness and that were embedded in images of real world scenes (as in Leder et al., 2010). We analyzed the mean number of fixations and total fixation duration to each of the more and the less attractive faces in the scenes. Additionally we gathered attractiveness ratings of the faces to have an additional, overt measure with which to compare the behavioral effects. We expected that: (1) participants would generally look longer at the attractive than the less attractive faces; (2) by including participants’ sexual orientation as a factor, looks at attractive faces should be longer for faces that match participants’ sexual orientation; (3) the attractiveness of faces matching participants’ sexual orientations is expected to have stronger effects for men—both heterosexual and homosexual—than for women, which means that men would look longer at the faces of their preferred sex than women, a prediction derived from the above studies (e.g., Alexander and Charles, 2009; Nummenmaa et al., 2012) showing that in general, attractiveness has a greater effect on men; and (4) the difference between the durations of looks at attractive and less attractive faces should be more pronounced for men than for women, and that heterosexual and homosexual women are expected to look at all faces similarly long.

## Materials and Methods

### Participants

Forty participants (20 men, 20 women; mean age, 23.38 years) participated in the study. Twenty participants were heterosexual (10 men, 10 women; mean age, 23.7 years) and were undergraduate students from the University of Vienna who participated for course credit. The 20 self-identified homosexual participants (10 men, 10 women; mean age, 23.05 years) consisted of both undergraduate students and participants recruited via the Internet (e.g., social networks, appropriate websites). The study was advertised as a visual perception study in which eye tracking will be used and that experimenters were looking for heterosexual and homosexual men and women who would like to participate. Prior to the start of the study, each participant reviewed and signed a consent form. Participants’ visual acuity, oculomotor dominance, color vision, and handedness were tested prior to the main study. All participants had normal or corrected-to-normal vision.

### Materials

**FIGURE 1:**
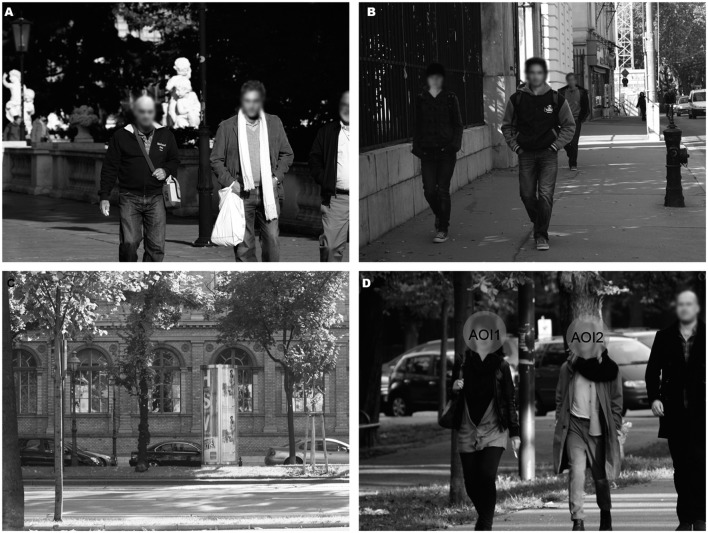
**Stimuli examples.** **(A)** Example of a same-sex scene showing a less attractive male (left) and attractive male (right) **(B)** Example of a filler scene showing a less attractive female (left) and attractive male (right) **(C)** Example of a filler scene without people **(D)** Example of a same-sex scene showing a less attractive female (left) and attractive female (right) with corresponding AOIs. Faces are blurred for reasons of anonymity.

To test our hypotheses, we used 35 images of real world scenes with each depicting two people of the same sex, with one of them being attractive and the other less attractive (17 pairs of women, 18 pairs of men; with attractiveness based on pre-study data as described below; see **Figure 1A**). In order to conceal the aim of the study and to create a natural “urban walk-like” sequence of scenes, the scenes were randomly mixed with eight filler scenes containing either an attractive woman paired with a less attractive man or vice versa (see **Figure 1B**). There were an additional 51 images of real world scenes without people, which were also used as filler scenes (as in Leder et al., 2010; see **Figure 1C**).

As in Leder et al. (2010), we replaced the faces in the original images of real world scenes with pre-selected and pre-rated attractive and less attractive faces of women and men. We wanted to use natural-looking stimuli and not isolated faces (as did e.g., Shimojo et al., 2003) in order to obtain a set of scenes that was more ecologically valid. In order to experimentally control for facial attractiveness, the original faces were replaced. This helped to ensure that one face was clearly more attractive than the other. To create the stimuli, we first collected a set of faces that would be used to replace the original faces in the scenes. Three people collected faces independently of each other. The following criteria guided the collection process: the individuals’ subjective evaluation of attractiveness (attractive, less attractive); faces with neutral expressions; faces with closed mouths and no teeth, smile, or facial jewelry visible; and for faces of men, no facial hair. To ensure that we had enough attractive and less attractive faces that would match the bodies in the original pictures, we collected a large pool of such faces. We performed two pre-studies to validate, and augment the rigor of, the initial face selection process as described above. In the first pre-study (*n* = 16), we established the attractiveness of the collected faces. Only faces that were clearly rated as attractive and less attractive were then used for the replacement of the original faces. In order to minimize the effects of clothes, height, and position, the faces were balanced over the left-right positions in the same-sex scenes (i.e., the test scenes). After the scenes were produced, we conducted the second pre-study (*n* = 16), which verified that the faces were still attractive (or less attractive) after being placed on the different bodies. In this second pre-study, participants saw both versions of each scene (attractive face on the left and less attractive face on the right, less attractive face on the left and attractive face on the right) and rated the attractiveness of each of the two people depicted and decided which of the two was more attractive. These pre-study participants rated a total of 58 same-sex scenes.

In the main study, we only included those 35 same-sex scenes from the pre-study in which the difference in attractiveness between the mean attractiveness ratings of the faces (within a scene) was at least 1.5 (on a 5-point-scale). Additionally, participants saw the above-described 59 filler scenes. Each participant in the main study viewed a total of 94 scenes.

### Procedure and Design

We employed a mixed-model design with the sexual orientation of the participants as the between-subject factor and the sex and attractiveness of the faces in the scenes as within-subject factors. The study consisted of two blocks. The first block involved a free-viewing task during which an EyeLink 1000 Desktop Mount eye tracker (SR Research Ltd., Mississauga, ON, Canada) recorded eye movements (left eye) at 1000 Hz frequency in a dimly lit room. The participants sat 62 cm away from the 24-inch Samsung SyncMaster 2443BW LCD monitor (widescreen; 16:9; resolution: 1920 × 1200 pixel; refresh rate 60 Hz) while forehead and chin rests stabilized their heads. The study was run on a computer using Windows XP and was controlled using Experiment Builder (SR Research Ltd.) software. Participants viewed all 94 scenes, with left-right positions counterbalanced between participants (59 filler and 35 same-sex scenes with 17 women and 18 men). The participants read the instructions, which stated that the aim of the study was to study visual perception behavior, that they should look at and explore the pictures freely as they wanted, and that there was no task involved. After the instructions were read, we performed a 9-point calibration check prior to each study. Each trial started with a fixation-cross in the middle of the screen. By fixating on the cross, the participant triggered the next stimulus. If there was no fixation during 5 s, the program showed an error message and a 5-point recalibration was performed. Each scene was presented for 10 s and participants’ eye movements were recorded during this period. We analyzed only fixations on the two faces for each scene and did not include the filler scenes in the analysis. We defined the areas covering each face as Areas of Interests (AOIs). AOIs were round in shape and covered each face area. The size of the AOIs was 100 pixels in diameter for all scenes (see **Figure 1D**). The dependent variables for this part of the study were the mean total fixation duration and mean number of fixations within the AOIs. We excluded blinks and saccades from the analysis.

The second block was used to further validate the attractiveness of the faces. In this block, participants rated the attractiveness of all of the faces that they saw in the first block. Participants sat in front of a 24-inch Samsung SyncMaster 2443BW LCD monitor (widescreen; 16:9; resolution: 1920 × 1200 pixel; refresh rate 60 Hz) and used a keyboard to input their ratings. After participants read the instructions stating that they will see the pictures that they saw before and that they should rate the attractiveness of the faces, participants viewed each same-sex scene twice. Whether the left or the right face was the first to be rated was randomized between participants. The participants provided ratings of all faces on a 7-point scale ranging from 1 (very unattractive) to 7 (very attractive). The mean attractiveness rating for each face served as the dependent variable in the second block.

Participants did not provide facial attractiveness ratings immediately after they explored the picture to ensure that their automatic, implicit response (first block) was isolated from the explicit responses to attractiveness that they provided during the evaluation task (second block). The presentation order of the scenes in the first and second blocks was randomized. After the study, participants completed a questionnaire regarding demographic data, relationship status, and sexual orientation. Finally, they were debriefed about the purpose of the study. The study was approved by the Ethics Committee of the University of Vienna.

## Results

The results are reported separately for the eye movement data and the attractiveness rating data. In all analyses reported, attractiveness (attractive, less attractive) and sex (faces of men and women, labeled as sex of face) of the embedded faces were within-subject factors, and sexual orientation (homosexual, heterosexual) of the participants was the between-subject factor. We did not include the participants’ relationship status as a factor in the analysis since the group sizes associated with this factor would have been too small. Throughout the results section, all pairwise comparisons are Bonferroni-corrected.

### Eye Movement Data

#### Mean Total Fixation Duration

**FIGURE 2:**
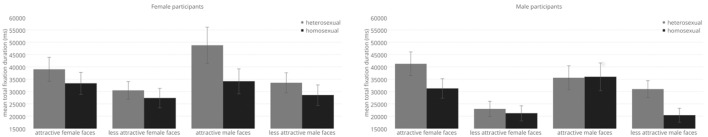
**Mean total fixation duration on attractive and less attractive female and male faces for heterosexual and homosexual women (left) and heterosexual and homosexual men (right).** Error bars represent ± 1 SE.

**Tables 1** and **2** show the means (“fixation duration”) sampled over participants separately for sex of participant (**Table 1** male, **Table 2** female participants). All analyses comprised a 2 (sexual orientation: heterosexual, homosexual) × 2 (sex of face: man, woman) × 2 (attractiveness: attractive, less attractive) mixed factorial repeated measures analysis of variance (ANOVA) design; the effect sizes for main effects and interactions of the reported ANOVAs are presented in **Tables 3**–**8.** The results are summarized in **Figure 2.** For the male participants, there was a significant main effect of attractiveness, *F*(1,18) = 40.09, *p* < 0.001, $\eta_{p}^{2}$ = 0.69 (see **Table 3**). Attractive faces were looked at longer than less attractive faces. There was also a significant interaction among attractiveness, sex of face, and sexual orientation, *F*(1,18) = 17.48, *p* < 0.01, $\eta_{p}^{2}$ = 0.50. *Post hoc* pairwise comparisons revealed that heterosexual men looked longer at less attractive faces of men than homosexual men (*p* < 0.05), that heterosexual men looked longer at attractive female faces than less attractive female faces (*p* < 0.001), that homosexual men looked longer at attractive female faces than less attractive female faces (*p* < 0.01), and that homosexual men looked longer at attractive male faces than less attractive male faces (*p* < 0.001). Furthermore, heterosexual men looked longer at less attractive male faces than less attractive female faces (*p* < 0.01), but they looked longer at attractive female faces than attractive male faces (*p* < 0.05). For the female participants, the same ANOVA revealed significant main effects of attractiveness, *F*(1,18) = 17.56, *p* < 0.01, $\eta_{p}^{2}$ = 0.49 and sex of face, *F*(1,18) = 5.32, *p* < 0.05, $\eta_{p}^{2}$ = 0.23 (see **Table 5**). Attractive faces were looked at longer than less attractive faces and male faces were looked at longer than female faces.

**Table 1:** Mean number of fixations (Fixation Count) and mean total fixation duration (Fixation Duration) on attractive and less attractive male and female faces and mean attractiveness ratings (Attractiveness rating) for attractive and less attractive male and female faces for heterosexual and homosexual men.

	Men	
	
	Heterosexual	Homosexual
		
	*M* (*SD*)	*M* (*SD*)
Fixation count				
Attractive male faces	81.10 (33.23)	76.10 (39.46)
Less attractive male faces	74.70 (30.20)	52.60 (26.37)
Attractive female faces	88.40 (35.84)	68.90 (31.64)
Less attractive female faces	61.40 (31.73)	55.50 (26.86)
Fixation duration				
Attractive male faces	35596.70 (15261.84)	35955.40 (17883.68)
Less attractive male faces	31009.50 (10805.90)	20391.20 (8992.44)
Attractive female faces	41265.00 (15143.47)	31246.40 (12483.55)
Less attractive female faces	22976.00 (9825.43)	21194.70 (9696.45)
Attractiveness rating				
Attractive male faces	4.59 (0.97)	5.17 (0.55)
Less attractive male faces	2.63 (0.45)	1.90 (0.45)
Attractive female faces	5.36 (0.59)	5.25 (0.53)
Less attractive female faces	2.80 (0.56)	2.69 (0.60)


**Table 2:** Mean number of fixations (Fixation Count) and mean total fixation duration (Fixation Duration) on attractive and less attractive male and female faces and mean attractiveness ratings (Attractiveness rating) for attractive and less attractive male and female faces for heterosexual and homosexual women.

	Women	
	
	Heterosexual	Homosexual
		
	*M* (*SD*)	*M* (*SD*)
Fixation count				
Attractive male faces	104.10 (48.43)	76.10 (35.65)
Less attractive male faces	80.60 (33.51)	68.20 (34.67)
Attractive female faces	85.50 (34.57)	71.90 (28.46)
Less attractive female faces	75.40 (26.94)	65.50 (30.27)
Fixation duration				
Attractive male faces	48769.10 (23160.61)	34143.00 (16008.71)
Less attractive male faces	33565.20 (12797.95)	28528.90 (13053.39)
Attractive female faces	39020.70 (15348.36)	33318.90 (14114.31)
Less attractive female faces	30491.20 (11295.57)	27356.10 (12432.03)
Attractiveness rating				
Attractive male faces	5.32 (0.61)	3.86 (1.00)
Less attractive male faces	2.27 (0.61)	1.88 (0.64)
Attractive female faces	5.64 (0.38)	4.54 (0.90)
Less attractive female faces	2.95 (0.52)	2.75 (0.86)


**Table 3:** Analysis of Variance for mean total fixation duration for male participants.

Source	df	*F*	η^2^	*p*
**Between subjects**				
Sexual orientation (SexOr)	1	1.12	0.06	0.30
Error	18			
**Within subjects**				
Attractiveness (A)	1	40.09^∗^	0.69	0.00
Sex of face (SexF)	1	2.31	0.11	0.15
A × SexF	1	3.24	0.15	0.09
A × SexOr	1	0.13	0.01	0.73
SexF × SexOr	1	0.14	0.01	0.71
A × SexF × SexOr	1	17.84^∗^	0.50	0.00
Error	18			


*^∗^*p* < 0.05.*

**Table 4:** Analysis of variance for mean number of fixations for male participants.

Source	df	*F*	η^2^	*p*
**Between subjects**				
Sexual orientation (SexOr)	1	0.90	0.05	0.36
Error	18			
**Within subjects**				
Attractiveness (A)	1	49.96^∗^	0.74	0.00
Sex of face (SexF)	1	1.41	0.07	0.25
A × SexF	1	1.77	0.09	0.20
A × SexOr	1	0.12	0.01	0.73
SexF × SexOr	1	0.04	0.00	0.85
A × SexF × SexOr	1	15.15^∗^	0.46	0.00
Error	18			


*^∗^*p* < 0.05.*

**Table 5:** Analysis of variance for mean total fixation duration for female participants.

Source	df	*F*	η^2^	*p*
**Between subjects**				
Sexual orientation (SexOr)	1	1.39	0.07	0.25
Error	18			
**Within subjects**				
Attractiveness (A)	1	17.56^∗^	0.49	0.00
Sex of face (SexF)	1	5.32^∗^	0.23	0.03
A × SexF	1	0.95	0.05	0.34
A × SexOr	1	2.08	0.10	0.17
SexF × SexOr	1	2.84	0.14	0.11
A × SexF × SexOr	1	1.18	0.06	0.29
Error	18			


*^∗^*p* < 0.05.*

**Table 6:** Analysis of variance for mean number of fixations for female participants.

Source	df	*F*	η^2^	*p*
**Between subjects**				
Sexual orientation (SexOr)	1	1.29	0.07	0.27
Error	18			
**Within subjects**				
Attractiveness (A)	1	8.26^∗^	0.31	0.01
Sex of face (SexF)	1	4.18	0.19	0.06
A × SexF	1	1.43	0.07	0.25
A × SexOr	1	1.34	0.07	0.26
SexF × SexOr	1	1.27	0.07	0.28
A × SexF × SexOr	1	0.91	0.05	0.35
Error	18			


*^∗^*p* < 0.05.*

**Table 7:** Analysis of variance for mean attractiveness ratings for male participants.

Source	df	*F*	η^2^	*p*
**Between subjects**				
Sexual orientation (SexOr)	1	0.47	0.03	0.50
Error	18			
**Within subjects**				
Attractiveness (A)	1	183.46^∗^	0.91	0.00
Sex of face (SexF)	1	16.34^∗^	0.48	0.00
A × SexF	1	0.13	0.01	0.73
A × SexOr	1	2.92	0.14	0.11
SexF × SexOr	1	0.02	0.00	0.89
A × SexF × SexOr	1	17.36^∗^	0.49	0.00
Error	18			


*^∗^*p* < 0.05.*

**Table 8:** Analysis of variance for mean attractiveness ratings for female participants.

Source	df	*F*	η^2^	*p*
**Between subjects**				
Sexual orientation (SexOr)	1	9.26^∗^	0.34	0.01
Error	18			
**Within subjects**				
Attractiveness (A)	1	371.84^∗^	0.95	0.00
Sex of face (SexF)	1	24.43^∗^	0.58	0.00
A × SexF	1	4.39	0.20	0.05
A × SexOr	1	16.01^∗^	0.47	0.00
SexF × SexOr	1	1.16	0.06	0.30
A × SexF × SexOr	1	0.43	0.02	0.52
Error				


*^∗^*p* < 0.05.*

#### Mean Number of Fixations

**Tables 1** and **2** present the means (“fixation count”) sampled over participants separately for sex of participant (**Table 1** male, **Table 2** female participants). Regarding the mean number of fixations, the ANOVA for the male participants revealed a significant main effect of attractiveness, *F*(1,18) = 49.96, *p* < 0.001, $\eta_{p}^{2}$ = 0.74 (see **Table 4**). Attractive faces were looked at more often than less attractive faces. The interaction amongst attractiveness, sex of face, and sexual orientation was also significant, *F*(1,18) = 15.15, *p* < 0.01, $\eta_{p}^{2}$ = 0.46. *Post hoc* pairwise comparisons revealed that heterosexual men looked more often at attractive female faces than less attractive female faces (*p* < 0.001), that homosexual men looked more often at attractive female faces than less attractive female faces (*p* < 0.01), and that homosexual men looked more often at attractive male faces than less attractive male faces (*p* < 0.001). And, heterosexual men looked more often at less attractive male faces than less attractive female faces (*p* < 0.01). For the female participants, the ANOVA only revealed a significant main effect of attractiveness, *F*(1,18) = 8.26, *p* < 0.05, $\eta_{p}^{2}$ = 0.31 (see **Table 6**). Attractive faces were looked at more often than less attractive faces.

### Behavioral Data

**Tables 1** and **2** also show the means of the attractiveness ratings sampled over participants (**Table 1** male, **Table 2** female participants). **Table 7** (male participants) and **Table 8** (female participants) show the effect sizes for the main effects and interactions of the reported ANOVAs. The ANOVA on mean attractiveness ratings for the male participants revealed significant main effects of attractiveness, *F*(1,18) = 183.46, *p* < 0.001, $\eta_{p}^{2}$ = 0.91, and sex of face, *F*(1,18) = 16.34, *p* < 0.01, $\eta_{p}^{2}$ = 0.48. Attractive faces were rated as more attractive than less attractive faces and female faces were rated as more attractive than male faces. There was a significant interaction amongst attractiveness, sex of face, and sexual orientation, *F*(1,18) = 17.36, *p* < 0.01, $\eta_{p}^{2}$ = 0.49. *Post hoc* pairwise comparisons revealed that heterosexual men rated less attractive male faces as more attractive than homosexual men (*p* < 0.01), that heterosexual men rated attractive female faces as more attractive than less attractive female faces (*p* < 0.001), that homosexual men rated attractive female faces as more attractive than less attractive female faces (*p* < 0.001), that heterosexual men rated attractive male faces as more attractive than less attractive male faces (*p* < 0.001), and that homosexual men rated attractive male faces as more attractive than less attractive male faces (*p* < 0.001). Furthermore, heterosexual men rated attractive female faces as more attractive than attractive male faces (*p* < .01). Furthermore, homosexual men rated less attractive female faces as more attractive than less attractive male faces (*p* < 0.001). For the female participants, the ANOVA revealed significant main effects of attractiveness, *F*(1,18) = 371.84, *p* < 0.001, $\eta_{p}^{2}$ = 0.95, sexual orientation, *F*(1,18) = 9.26, *p* < 0.01, $\eta_{p}^{2}$ = 0.34, and sex of face, *F*(1,18) = 24.43, *p* < 0.001, $\eta_{p}^{2}$ = 0.58. Attractive faces were generally rated as more attractive than less attractive faces, heterosexual women rated faces as more attractive than homosexual women, and female faces were rated as more attractive than male faces. There was a significant interaction between attractiveness and sexual orientation, *F*(1,18) = 16.01, *p* < 0.01, $\eta_{p}^{2}$ = 0.47. *Post hoc* pairwise comparisons revealed that heterosexual women rated attractive faces as more attractive than homosexual women (*p* < 0.001), that heterosexual women rated attractive faces as more attractive than less attractive faces (*p* < 0.001), and that homosexual women rated faces as more attractive than less attractive faces (*p* < 0.001).

## Discussion

Facial attractiveness plays an important role during our interactions with others in the world. While it has been demonstrated that we look longer at attractive people, it has not been shown how this effect is associated with sexual orientation, in particular when the to be rated faces or persons are seen in a natural context. We examined the visual perception and evaluation—through measures of eye movements and attractiveness ratings—of heterosexual men and women and homosexual men and women toward images of attractive and less attractive men and women embedded in images of real world, urban street scenes.

The visual perception and evaluation of faces varying in attractiveness converged in this study. Pre-selected attractive faces, independent of their sex, were looked at longer and more often, and were rated as more attractive, as compared to less attractive faces. In nearly all comparisons, the faces pre-selected as attractive were rated as significantly more attractive and were also looked at significantly more often and longer than the less attractive faces. Despite an overall strong tendency to look longer at attractive faces, we also discovered systematic variations in the way attractiveness draws longer looks in participants with different sexual orientations.

What did the examination of participants’ eye movements reveal about the relative importance of sexual orientation? We found that of the three main factors in the study (facial attractiveness, sex of the face, and participants’ sexual orientation), level of attractiveness was most relevant, as indicated by the fact that participants looked longer and more often at attractive faces than less attractive faces. This is a result that we had expected. However, sexual orientation had a critical moderating effect, as indicated by the finding that participants—except for homosexual women—looked longer and more often at attractive faces that matched their sexual orientations than faces of the other sex. For homosexual women, the differences between sexually preferred and non-preferred faces were quite small.

In contrast to homosexual men and heterosexual women, heterosexual men and homosexual women did not look longer at attractive male faces as compared to less attractive male faces (see **Tables 1** and **2**). The interactions in both dependent measures (mean number of fixations, mean total fixation duration) indicate that responses toward attractiveness for male participants varied as a function of sexual orientation. Interestingly, heterosexual and homosexual men and homosexual women looked longer and more often at the less attractive face of their non-preferred sex than the less attractive face of their preferred sex. It seems that faces that are less attractive, but of the preferred sex, have an aversive character and therefore received the least amount of attention. This finding is consistent with the findings of, for example, Kampe et al. (2001) and Cloutier et al. (2008). When less attractive faces were presented Cloutier et al. (2008) found activation in the lateral orbitofrontal cortex, a region that is associated with punishment (see O’Doherty et al., 2001). Heterosexual men looked longer and more often at attractive female faces as compared to less attractive female faces and also as compared to attractive male faces, a finding that is consistent with their sexual orientation. Also consistent is the finding that male faces and their attractiveness are less important to heterosexual men (the differences between attractive and less attractive male faces for both dependent measures were not significant in *post hoc* pairwise comparisons). A possible explanation of why heterosexual men looked similarly long at the two types of male faces is intra-sexual comparison: heterosexual men may have compared themselves with the men in the pictures. Heterosexual men differed from homosexual men and heterosexual women in this regard with the latter two groups both showing clear differences in looking behavior between attractive and less attractive male and female faces. These latter effects correspond to the sexual orientations of homosexual men and heterosexual women. Heterosexual women looked longer at attractive male faces than attractive female faces. Although this finding corresponds to heterosexual women’s sexual orientation, it contrasts the results of Leder et al. (2010), who found that women looked longer at female faces as compared to male faces and that women looked longest at attractive female faces.

Attractiveness affected homosexual women least, as this group of participants did not look more often or longer at attractive male faces and not more often at attractive female faces as compared to the less attractive counterparts. This is in accordance with the results of Bailey et al. (1994) and Ha et al. (2012). For homosexual women, in comparison to the other participant groups, the differences between attractive and less attractive faces, regardless of the sex of the face, were much smaller—only about half the size. However, when looking at two female faces, homosexual women looked longer at the attractive female face. Interestingly, and somehow unexpectedly, homosexual women looked longer and more often at male faces as compared to female faces when attractiveness is not taken into account. This can be seen in **Table 2** when combining the values of less attractive and more attractive male faces and comparing them to the combination of less attractive and more attractive female faces. What are possible explanations for this finding? Visual attention toward men could indeed be a sign of some biological interest associated with a wish to reproduce. Alternatively, this effect might reflect cautious behavior toward men, who are generally seen as more aggressive and threatening than women (Leder et al., 2010, Experiment 2). However, if this latter explanation were correct, then attractive and less attractive male faces would have received similar amounts of attention. This was not the case. Thus, while this effect might indicate a biological wish for reproduction, the effects of attractiveness for homosexual women are—as expected—rather weak; the differences in the dependent variables between attractive and less attractive faces are small (Bailey et al., 1994). The differences between attractive and less attractive faces are smaller compared to the other participant groups.

When analyzing participants’ attractiveness ratings of one attractive and one less attractive face in scenes depicting two people of the same sex, we found that in general, attractive faces were rated as more attractive as compared to less attractive faces. This serves as a manipulation check and shows that our experimental manipulation succeeded. With respect to participants’ sexual orientation, it can be said that the following findings associated with homosexual women and heterosexual men are indicative of the dominant effects of sexual orientation: homosexual women rated faces matching their sexual orientation; that is, they rated female faces as more attractive than male faces, regardless of the level of facial attractiveness. Similarly, heterosexual men rated faces matching their sexual orientation. This pattern, which was also shown by heterosexual women and homosexual men, is unexpected for these groups of participants because it does not match their sexual orientation; however, it can be said that heterosexual women’s ratings are in accordance with Leder et al. (2010).

It is interesting that heterosexual men rated less attractive male faces as more attractive as compared to homosexual men. Consistent with this finding is that homosexual men rated less attractive female faces as more attractive as compared to less attractive male faces. Again, it seems that less attractive faces that match the participants’ sexual orientation somehow seemed aversive.

Thus, our data show how sexual orientation affected behavior. The hypothesis that attractive faces receive more attention than less attractive faces was also generally supported. Our findings are also in accordance with previous findings that attractiveness is less important to homosexual women (Bailey et al., 1994; Ha et al., 2012).

A limitation of our study is that we determined participants’ sexual orientation categorically instead of using a scale. It cannot be ruled out that some of our (self-identified) heterosexual and homosexual participants could have tended more toward bisexuality. However, the fact that the homosexual participants indicated their sexual orientation as homosexual, and the fact that we found effects consistent with these classifications, lend some support for the validity of our approach.

Other variables with additional explanatory value, and that could be the focus of future research, are the participants’ relationship status and their desire to have children. Future studies could focus on sexual attractiveness, but not general attractiveness as this study did. A heterosexual man could rate another man as attractive—in the sense of general appearance. This does not necessarily mean he is sexually attracted to him. From a Darwinian standpoint, one would expect the differences in such attractiveness ratings for female and male faces to be more pronounced as found in this study. Heterosexual men, and to a lesser degree homosexual women, should clearly prefer female faces, whereas homosexual men and heterosexual women should clearly prefer male faces. Nevertheless, sexually non-preferred faces that are attractive should receive higher ratings from homosexual individuals because homosexual individuals are also interested in having children (Wyers, 1987; Mondimore, 1996; Lippa, 2007; Gates, 2008, 2013a,b). However, disentangling these factors is difficult within the context of the present study.

The present study suggests that our visual exploratory behavior is strongly determined by beauty. Beauty demands longer looks, and indeed, attractive faces received longer looks. But when it comes to such behaviors, our findings emphasize the importance of sexual orientation. It could thus be assumed that the processing of attractiveness manifests itself through a combination of sexual orientation and the specific motivations and goals of an individual. Sexual orientation directly affects the processing of attractiveness and is aligned with visual behavior. Our findings are relevant for the design of studies in which facial attractiveness, and its consequences on the perceiver, are addressed. Ignoring inter-individual differences, such as the sexual orientation, might reveal an overly simplistic image. Finally, our use of real-world scenes demonstrates that tight experimental control could be successfully balanced with ecological validity.

## Author Contributions

AM was involved in the planning of the design of the experiment, created a part of the used stimuli, collected, analyzed and interpreted the data. She wrote the manuscript and revised it. HL was involved in the planning of the design and interpretation of the results, wrote parts of and revised the manuscript. He gave his approval of the version to be published. PT was also involved in the planning of the design, created a part of the used stimuli, and helped with the interpretation of the data. He wrote parts of the manuscript and revised it. He also gave his approval of the version to be published.

## Conflict of Interest Statement

The authors declare that the research was conducted in the absence of any commercial or financial relationships that could be construed as a potential conflict of interest.

## Footnotes

1 Throughout the manuscript, we will omit the use “pre-selected” to maintain the flow of reading. Each mention of attractive or less attractive faces are based on pre-studies that established the levels of attractiveness.

## References

[B1] Alexander G. M. Charles N. (2009). Sex differences in adults’ relative visual interest in female and male faces, toys, and play styles. *Arch. Sex. Behav.* 38 434–441. 10.1007/s10508-008-9429-7 19016319

[B2] Bailey J. M. Gaulin S. Agyei Y. Gladue B. (1994). Effects of gender and sexual orientation on evolutionary relevant aspects of human mating psychology. *J. Pers. Soc. Psychol.* 66 1081–1093. 10.1037/0022-3514.66.6.1081 8046578

[B3] Cloutier J. Heatherton T. F. Whalen P. J. Kelley W. M. (2008). Are attractive people rewarding? Sex differences in the neural substrates of facial attractiveness. *J. Cogn. Neurosci.* 20 941–951. 10.1162/jocn.2008.20062 18211242 PMC3848031

[B4] Darwin C. (1871). *The Descent of Man, and Selection in Relation to Sex.* London: Murray.

[B5] Dion K. Berscheid E. Walster E. (1972). What is beautiful is good. *J. Pers. Soc. Psychol.* 24 285–290. 10.1037/h0033731 4655540

[B6] Dissanayake E. (2007). “What art is and what art does: An overview of contemporary evolutionary hypotheses,” in *Evolutionary and Neurocognitive Approaches to Aesthetics, Creativity and the Arts*, eds Martindale C. Locher P. Petrov V. M. (Amityville, NY: Baywood), 1–14.

[B7] Fromberger P. Jordan K. von Herder J. Steinkrauss H. Nemetschek R. Stolpmann G. (2011). Initial orienting towards sexually relevant stimuli: preliminary evidence from eye movement measures. *Arch. Sex. Behav.* 41 919–928. 10.1007/s10508-011-9816-3 21792688 PMC3394233

[B8] Gates G. (2008). “Diversity among same-sex couples and their children,” in *American Families: A Multicultural Reader*, ed. Coontz S. (New York, NY: Routledge), 394–399.

[B9] Gates G. J. (2013a). *Same-sex and Different-sex Couples in the American Community Survey: 2005–2011.* Available at: http://williamsinstitute.law.ucla.edu/wp-content/uploads/ACS-2013.pdf

[B10] Gates G. J. (2013b). *LGBT Parenting in the United States.* Available at: http://williamsinstitute.law.ucla.edu/wp-content/uploads/LGBT-Parenting.pdf

[B11] Ha T. van den Berg J. E. Engels R. C. Lichtwarck-Aschoff A. (2012). Effects of attractiveness and status in dating desire in homosexual and heterosexual men and women. *Arch. Sex. Behav.* 41 673–682. 10.1007/s10508-011-9855-9 21979410

[B12] Ishai A. (2007). Sex, beauty and the orbitofrontal cortex. *Int. J. Psychophysiol.* 63 181–185. 10.1016/j.ijpsycho.2006.03.010 16759727

[B13] Israel E. Strassberg D. S. (2009). Viewing time as an objective measure of sexual interest in heterosexual men and women. *Arch. Sex. Behav.* 38 551–558. 10.1007/s10508-007-9246-4 17943432

[B14] Kampe K. K. Frith C. D. Dolan R. J. Frith U. (2001). Reward value of attractiveness and gaze. *Nature* 413:589 10.1038/35098149 11595937

[B15] Kranz F. Ishai A. (2006). Face perception is modulated by sexual preference. *Curr. Biol.* 16 63–68. 10.1016/j.cub.2005.10.070 16401423

[B16] Leder H. Tinio P. P. L. Fuchs I. Bohrn I. (2010). When attractiveness demands longer looks: the effects of situation and gender. *Q. J. Exp. Psychol.* 63 1858–1871. 10.1080/17470211003605142 20373226

[B17] LeVay S. (1991). A difference in hypothalamic structure between heterosexual and homosexual men. *Science* 253 1034–1037. 10.1126/science.1887219 1887219

[B18] Lippa R. A. (2007). The preferred traits of mates in a cross-national study of heterosexual and homosexual men and women: an examination of biological and cultural influences. *Arch. Sex. Behav.* 36 193–208. 10.1007/s10508-006-9151-2 17380374

[B19] Lippa R. A. (2012). Effects of sex and sexual orientation on self-reported attraction and viewing times to images of men and women: testing for Category Specificity. *Arch. Sex. Behav.* 41 149–160. 10.1007/s10508-011-9898-y 22258278

[B20] Lykins A. D. Meana M. Strauss G. P. (2008). Sex differences in visual attention to erotic and non-erotic stimuli. *Arch. Sex. Behav.* 37 219–228. 10.1007/s10508-007-9208-x 17668312

[B21] Mondimore F. M. (1996). *A Natural History of Homosexuality.* Baltimore: The John Hopkins University Press.

[B22] Nummenmaa L. Hietanen J. K. Santtila P. Hyönä J. (2012). Gender and visibility of sexual cues influence eye movements while viewing faces and bodies. *Arch. Sex. Behav.* 41 1439–1451. 10.1007/s10508-012-9911-0 22402995

[B23] O’Doherty J. Kringelbach M. L. Rolls E. T. Hornak J. Andrews C. (2001). Abstract reward and punishment representations in the human orbitofrontal cortex. *Nat. Neurosci.* 4 95–102. 10.1038/82959 11135651

[B24] Russock H. I. (2011). An evolutionary interpretation of the effect of gender and sexual orientation on human mate selection preferences, as indicated by an analysis of personal advertisements. *Behaviour* 148 307–323. 10.1163/000579511X556600

[B25] Savic I. Lindström P. (2008). PET and MRI show differences in cerebral asymmetry and functional connectivity between homo- and heterosexual subjects. *Proc. Natl. Acad. Sci. U.S.A.* 105 9403–9408. 10.1073/pnas.0801566105 18559854 PMC2453705

[B26] Senior C. (2003). Beauty in the brain of the beholder. *Neuron* 38 525–528. 10.1016/S0896-6273(03)00293-9 12765605

[B27] Shimojo S. Simion C. Shimojo E. Scheier C. (2003). Gaze bias both reflects and influences preference. *Nat. Neurosci.* 6 1317–1322. 10.1038/nn1150 14608360

[B28] Swaab D. F. Hofman M. A. (1990). An enlarged suprachiasmatic nucleus in homosexual men. *Brain Res.* 537 141–148. 10.1016/0006-8993(90)90350-K 2085769

[B29] Thornhill R. Gangestad S. W. (1993). Human facial beauty: averageness, symmetry, and parasite resistance. *Hum. Nat.* 4 237–269. 10.1007/BF02692201 24214366

[B30] Thornhill R. Gangestad S. W. (1996). The evolution of human sexuality. *Trends Ecol. Evol.* 11 98–102. 10.1016/0169-5347(96)81051-2 21237770

[B31] Thornhill R. Grammer K. (1999). The body and face of woman: one ornament that signals quality? *Evol. Hum. Behav.* 20 105–120. 10.1016/S1090-5138(98)00044-0

[B32] Wyers N. L. (1987). Homosexuality in the family: lesbian and gay spouses. *Soc. Work* 32 143–148.

